# The activation of adenosine monophosphate–activated protein kinase inhibits the migration of tongue squamous cell carcinoma cells by targeting Claudin‐1 via epithelial–mesenchymal transition

**DOI:** 10.1002/ame2.12444

**Published:** 2024-07-17

**Authors:** Xin‐Yue Zhou, Qiu‐Ming Liu, Zhuang Li, Xia‐Yang Liu, Qi‐Wei Zhao, Yu Wang, Feng‐Hua Wu, Gang Zhao, Rui Sun, Xiao‐Hong Guo

**Affiliations:** ^1^ Department of Basic Medicine Hubei University of Chinese Medicine Wuhan China; ^2^ Hubei Shizhen Laboratory Wuhan Hubei China; ^3^ Sino‐German Biomedical Center Hubei University of Technology Wuhan China; ^4^ Center of Applied Biotechnology Wuhan Institute of Bioengineering Wuhan China; ^5^ Department of Stomatology, Shanxi Bethune Hospital, Shanxi Academy of Medical Sciences, Tongji Shanxi Hospital Third Hospital of Shanxi Medical University Taiyuan China; ^6^ Department of Oral and Maxillofacial Surgery Shanxi Provincial People's Hospital Taiyuan China

**Keywords:** AMPK, Claudin‐1, EMT, migration, tongue squamous cell carcinoma

## Abstract

**Background:**

The role of Claudin‐1 in tongue squamous cell carcinoma (TSCC) metastasis needs further clarification, particularly its impact on cell migration. Herein, our study aims to investigate the role of Claudin‐1 in TSCC cell migration and its underlying mechanisms.

**Methods:**

36 TSCC tissue samples underwent immunohistochemical staining for Claudin‐1. Western blotting and immunofluorescence analyses were conducted to evaluate Claudin‐1 expression and distribution in TSCC cells. Claudin‐1 knockdown cell lines were established using short hairpin RNA transfection. Migration effects were assessed through wound healing assays. Furthermore, the expression of EMT‐associated molecules was measured via western blotting.

**Results:**

Claudin‐1 expression decreased as TSCC malignancy increased. Adenosine monophosphate–activated protein kinase (AMPK) activation led to increased Claudin‐1 expression and membrane translocation, inhibiting TSCC cell migration and epithelial–mesenchymal transition (EMT). Conversely, Claudin‐1 knockdown reversed these inhibitory effects on migration and EMT caused by AMPK activation.

**Conclusions:**

Our results indicated that AMPK activation suppresses TSCC cell migration by targeting Claudin‐1 and EMT pathways.

## Graphical abstract

Schematic diagram of the potential antitumor mechanism of adenosine monophosphate–activated protein kinase (AMPK) in tongue squamous cell carcinoma (TSCC) cells. The activation of AMPK by 5‐aminoimidazole‐4‐carboxamide1‐β‐d‐ribofuranoside (AICAR) increased Claudin‐1 expression, induced its membrane translocation, and inhibited the EMT and migration of TSCC cells. Moreover, the knockdown of Claudin‐1 reversed the inhibitory effects of AMPK on EMT and migration. This cascade of events implies that the possible mechanism of the inhibitory effects of AMPK on the migration of tongue squamous cell carcinoma TSCC cells was mediated by Claudin‐1 via the suppression of EMT.

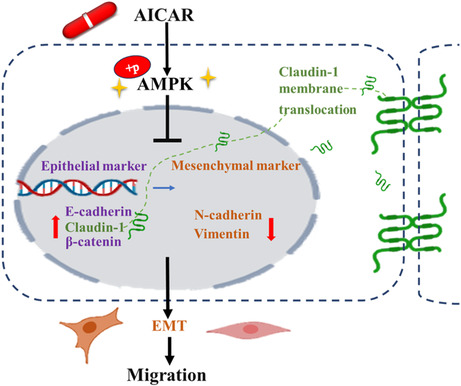

## 1 INTRODUCTION

Tongue squamous cell carcinoma (TSCC) is the most common oral cancer in Southeast Asia^1^ and is characterized by high mortality rates, rapid growth, strong invasiveness, early locoregional lymph node metastasis, poor prognosis, and serious harm to human health. Presently, the principal treatment for TSCC is surgery, followed by chemotherapy, radiotherapy, targeted therapy, and immunotherapy, all of which have notable side effects.^2^ Therefore, identifying the underlying mechanisms of tongue cancer and exploring effective preventive methods and therapeutic drugs is crucial and warranted.

The occurrence, development, and metastasis of tumors are multifactorial processes that include primary cancer growth, invasion and migration, clonal proliferation, angiogenesis, and other processes.^3^, ^4^ Tight junctions (TJs) are dynamic structures located at the joint interface between epithelial and endothelial cells.^5^ Many studies have confirmed that TJs are the first hurdle in tumor cell metastasis. To metastasize, tumor cells must lose their intercellular adhesion and separate from the TJ structure.^6^, ^7^ Claudins comprise four transmembrane regions, with molecular weights ranging from 20 to 34 kDa, which play important roles in maintaining the integrity of TJ by regulating paracellular barrier permeability and in cell proliferation and differentiation by interacting with other signaling molecules.^8^ Previous studies have shown that Claudin‐1 exhibits differential expression patterns and exerts direct regulatory effects, acting as a tumor promoter, suppressor, or both, depending on the tumor type.^7^, ^9^ For example, in colorectal adenocarcinoma^10^ and oral squamous cell carcinoma (OSCC),^11^, ^12^ Claudin‐1 acts as a tumor promoter and promotes the tumor cell migration. However, in human cervical squamous cell carcinoma^13^ and TSCC,^14^ Claudin‐1 acts as a tumor suppressor, and the knockdown of Claudin‐1 can considerably promote the proliferation and migration of the tumor cells. Claudin‐1 expression is associated with tumor malignancy. Claudin‐1 is strongly expressed in the precancerous stage, and its expression gradually decreases during progression to the invasive stage in cervical squamous intraepithelial neoplasia and invasive carcinoma.^15^ Additionally, Claudin‐1 is highly expressed in well‐differentiated OSCC, whereas it is predominantly absent in poorly differentiated OSCC.^12^ In summary, the expression and function of Claudin‐1 in squamous cell carcinoma (SCC) remain unclear.

Epithelial–mesenchymal transition (EMT) is a process in which cells switch from epithelial to mesenchymal characteristics, following the downregulation of the epithelial marker E‐cadherin and the upregulation of the mesenchymal marker vimentin. EMT is involved in various biological functions in malignancies, including tumor initiation, progression, invasive migration, and drug resistance.^16^ Adenosine monophosphate–activated protein kinase (AMPK) is a ubiquitous serine/threonine kinase found in eukaryotic cells that functions as a cellular energy receptor and metabolic regulator and has been proven to be involved in regulating the proliferation, invasion, and migration of tumor cells.^17^ Moreover, AMPK activation can specifically alter the early phases of TJ assembly, and the phosphorylation of AMPK can increase the translocation of the cytoplasmic protein zonula occludens‐1 (ZO‐1) to the cell membrane.^18^ This effect was initially observed in the Madin–Darby canine kidney cells, where AMPK inhibition impeded the TJ reassembly, whereas AMPK activation promoted the TJ assembly, thereby facilitating the migration of ZO‐1.^19^ Additionally, we previously demonstrated that AMPK activation could induce ZO‐1 translocation to the membrane and inhibit the migration of TSCC cells.^20^ AMPK activation significantly inhibits EMT in tumor cells. For example, AMPK activation by capsaicin inhibited migration, invasion, and EMT in renal cancer cells.^21^ Moreover, AMPK activation by 5‐aminoimidazole‐4‐carboxamide1‐β‐d‐ribofuranoside (AICAR) inhibited the migration and EMT of pancreatic cancer cells, increased the expression of E‐cadherin, and downregulated the expression of vimentin.^22^ However, the potential relationship among Claudin‐1, EMT, and AMPK in TSCC has not been fully elucidated.

Herein, we investigated the expression of Claudin‐1 in cancer tissues from 36 patients with TSCC and explored the effects of AMPK on the expression and distribution of Claudin‐1, EMT, and cell migration in vitro. We also evaluated the role of Claudin‐1 in the inhibitory effects of AMPK on EMT and cell migration.

## 2 MATERIALS AND METHODS

### 2.1 Antibody reagents

Rabbit anti‐Claudin‐1 polyclonal antibody (BS6778, 1:2000) and rabbit anti‐glyceraldehyde 3‐phosphate dehydrogenase (GAPDH) polyclonal antibody (AP0066, 1:10 000) were purchased from Bioworld Technology (Saint Louis Park, MN, USA); rabbit anti‐AMPK polyclonal antibody (AF6423, 1:2000) and rabbit anti‐phospho‐AMPK Thr172 polyclonal antibody (AF3423, 1:2000) were purchased from Affinity Biosciences (Melbourne, Australia). Both rabbit anti‐E‐cadherin polyclonal antibodies (20874‐1‐AP, 1:20 000), rabbit anti‐N‐cadherin polyclonal antibody (22018‐1‐AP, 1:10 000), rabbit anti‐vimentin polyclonal antibody (10366‐1‐AP, 1:10 000), and rabbit anti‐β‐catenin polyclonal antibody (51067‐2‐AP, 1:6000) were obtained from Proteintech Group (Wuhan, China).

### 2.2 Clinical tissue specimens

Tongue tissue samples were surgically removed from 36 individuals diagnosed with TSCC at the Stomatology School of Peking University in Beijing, China. The study participants consisted of 20 men and 16 women, ranging in age from 27 to 76 years, with a median age of 67. None of the patients underwent radiation therapy, chemotherapy, or other cancer treatments. The samples were obtained from areas located a minimum of 2 cm away from the tumor and were processed and categorized into cancerous and adjacent noncancerous tissues. The Ethics Committee of Peking University Health Science Center (IRB00001052‐09087) granted approval for this clinical investigation. Before enrolling in the study, all patients provided informed written consent.

### 2.3 Immunohistochemistry

The clinical tissues were embedded in paraffin and then cut into 4‐μm sections. Before immunostaining, the sections were dewaxed in xylene I and xylene II (Sinopharm Chemical Reagent, Shanghai, China) for each 10 min, rehydrated in graded ethanol (Sinopharm Chemical Reagent), and washed thrice with distilled water for 5 min. The sections were then placed in an antigen retrieval box, submerged in a citrate‐based solution (Sinopharm Chemical Reagent, China), and microwaved for 15 min. Then, the sections were incubated with the anti‐Claudin‐1 antibody (BS6778; 1:50; Bioworld Technology) overnight at 4°C. Following this, the sections were treated with a secondary antibody for 50 min using the two‐step ultrasensitive immunohistochemical detection reagent (ZSGB‐BIO, Beijing, China) before staining with 3–3′‐diaminobenzidine tetrahydrochloride (DAB) from the DAB Kit (ZSGB‐BIO). Next, the sections were observed under a microscope, and at least five random fields of view were selected for the analysis. Finally, the immunohistochemistry (IHC) positive index (PI), cumulative optical density value, and positive pixel area were analyzed using Image‐Pro Plus 6.0. The PI was chosen from the average of at least five different vision levels and calculated using the following formula:

$$\mathrm{PI}\;\left(\%\right)=\left(\text{mean optical density of positive area}/\text{positive area}\right)\times 100$$

### 2.4 Cell culture and transfection

Human TSCC cell lines SCC9 and Cal27 were procured from the American Type Culture Collection (ATCC; Manassas, VA, USA). SCC9 cells were grown in Dulbecco's modified Eagle medium (DMEM)/nutrient mixture F‐12 (Thermo Fisher Scientific, USA) supplemented with 10% fetal bovine serum (FBS, Excell), 100 U/mL penicillin, 100 μg/mL streptomycin (Hyclone, USA), 1.2 g/L sodium bicarbonate, 0.5 mmol/L sodium pyruvate (Biosharp, Hefei, China), and 400 ng/mL hydrocortisone (BBI Life Science, China). On the contrary, Cal27 cells were cultured in DMEM (Thermo Fisher Scientific, Waltham, MA, USA) with 10% FBS (Excell), 100 U/mL penicillin, and 100 μg/mL streptomycin (Hyclone, Omaha, NE, USA). The cells were incubated at 37°C in a humidified incubator with 5% CO_2_. An AMPK inhibitor, AICAR (A9978; Sigma‐Aldrich, St. Louis, MO, USA), was diluted in sterile water and kept at −20°C. The cells were exposed to varying concentrations of AICAR as per the experimental layout, with untreated cells serving as the control group. A lentiviral expression vector, namely pLenti–short hairpin RNA (shRNA)–green fluorescent protein (GFP)–Puro, psPAX2 (lentivirus packaging plasmid), pMD2G (lentivirus envelope plasmid), and shRNAs targeted against Claudin‐1 messenger RNA (mRNA) were constructed by Bioeagle Technologies (Wuhan, China). The plasmid vector containing the GFP gene was used to directly observe transfection efficiency. To construct the lentivirus, psPAX2 and pMD2G were cotransfected into 293 T cells using liposomes (Neofect, Beijing, China) with a lentiviral expression vector, such as a vehicle vector (shCon) or three different shRNA‐targeting plasmids that interfere with Claudin‐1 expression (namely sh1, sh2, and sh3). Claudin‐1 shRNAs include sh1: 5′‐GCAATCTTTGTGGCCACCGTT‐3′; sh2: 5′‐GCAATAGAATCGTTCAAGA‐3′; and sh3: 5′‐GTCTTTGACTCCTTGCTGA‐3′ sequences. After 24–48 h of transfection, the virus supernatant was harvested and stored at −80°C. Subsequently, 500 μL of the virus supernatant mixed with 1.5 mL of normal complete medium was used to infect SCC9 cells. After 48 h of infection, the cells were selected by 2.0 μg/mL puromycin for 2–3 weeks and then were subjected to western blotting.

### 2.5 Immunofluorescence

Cells were seeded on glass slides for either 24 or 48 h before being treated with 1 mmol/L AICAR. Cell coverage was provided using additional glass slides during the treatment process. Following treatment, cells underwent three washes with phosphate‐buffered saline (PBS) and were subsequently immersed in a 4% paraformaldehyde solution for 20 min. Subsequent steps included additional three washes with PBS, permeabilization with 0.1% Triton X‐100 (BL934B, Biosharp) for 10 min, blocking with 5% bovine serum albumin for 30 min, and overnight incubation at 4°C with an anti‐Claudin‐1 antibody (dilution ratio 1:50, BS6778, Bioworld Technology). After further three 5‐min washes with PBS, cells were incubated with an FITC‐labeled secondary antibody (BS22349, Biosharp) for 2 h at 37°C. The final step involved analysis of fluorescence images using an inverted fluorescence microscope (Olympus Corporation, model IX71, Tokyo, Japan).

### 2.6 Western blotting

To extract the total protein, cells were collected, washed in ice‐cold PBS, and lysed in a buffer containing protease and phosphatase inhibitors (Biosharp, China) for 30 min on ice. The lysates were then centrifuged for 15 min at 4°C and 12 000 rpm/min, and the supernatant was collected in a new tube on ice. Subsequently, the bicinchoninic acid (BCA) protein assay kit (YEASEN, Shanghai, China) was used to determine the protein concentrations. After denaturation, the proteins (approximately 20 μg/lane) were separated by sodium dodecyl sulfate–polyacrylamide gel electrophoresis (SDS‐PAGE), electrically transferred to a polyvinylidene fluoride (PVDF) membrane, blocked using 5% non‐fat dry milk in tris‐buffered saline–Tween 20 (TBST) for 2 h, and washed five times with TBST for 5 min. Subsequently, the membranes were probed with corresponding primary antibodies overnight at 4°C, washed five times with TBST, and covered with horse–radish peroxidase‐conjugated secondary antibody at room temperature for 1 h. Finally, we used enhanced chemiluminescence (Biosharp) and chemiluminescence imaging system (Bio‐Rad, Boulder, CO, USA) to visualize the proteins. ImageJ 2.3.0 was used to quantify proteins, and each assay was performed in triplicate.

### 2.7 Wound healing assays

Cells were plated in six‐well dishes and incubated for 24 h, allowing them to grow until reached 90% confluency, before being harvested. Scratch wounds were created in the cell monolayers using a sterile pipette tip, followed by three washes with PBS to eliminate any unattached cells. The growth medium was then substituted with 1% FBS supplemented with 1 mmol/L AICAR. The progress of wound closure was documented at both 0 and 48 h post‐scratch, and cell migration was quantified using ImageJ software with triplicate trials for each experiment.

### 2.8 Statistical analysis

The mean ± standard deviation data were presented. An analysis of variance was conducted using the one‐way method, followed by the Tukey post hoc test to examine variances among multiple groups. Two‐tailed Student's *t*‐tests, either paired or unpaired, were conducted to compare differences between two groups with comparable variances. Statistical analysis was performed utilizing GraphPad Prism 5.0 and Statistical Package for Social Sciences 26.0. A *p*‐value <0.05 indicated statistical significance.

## 3 RESULTS

### 3.1 The expression of Claudin‐1 decreased with the increase in the clinical stage of TSCC

**FIGURE 1:**
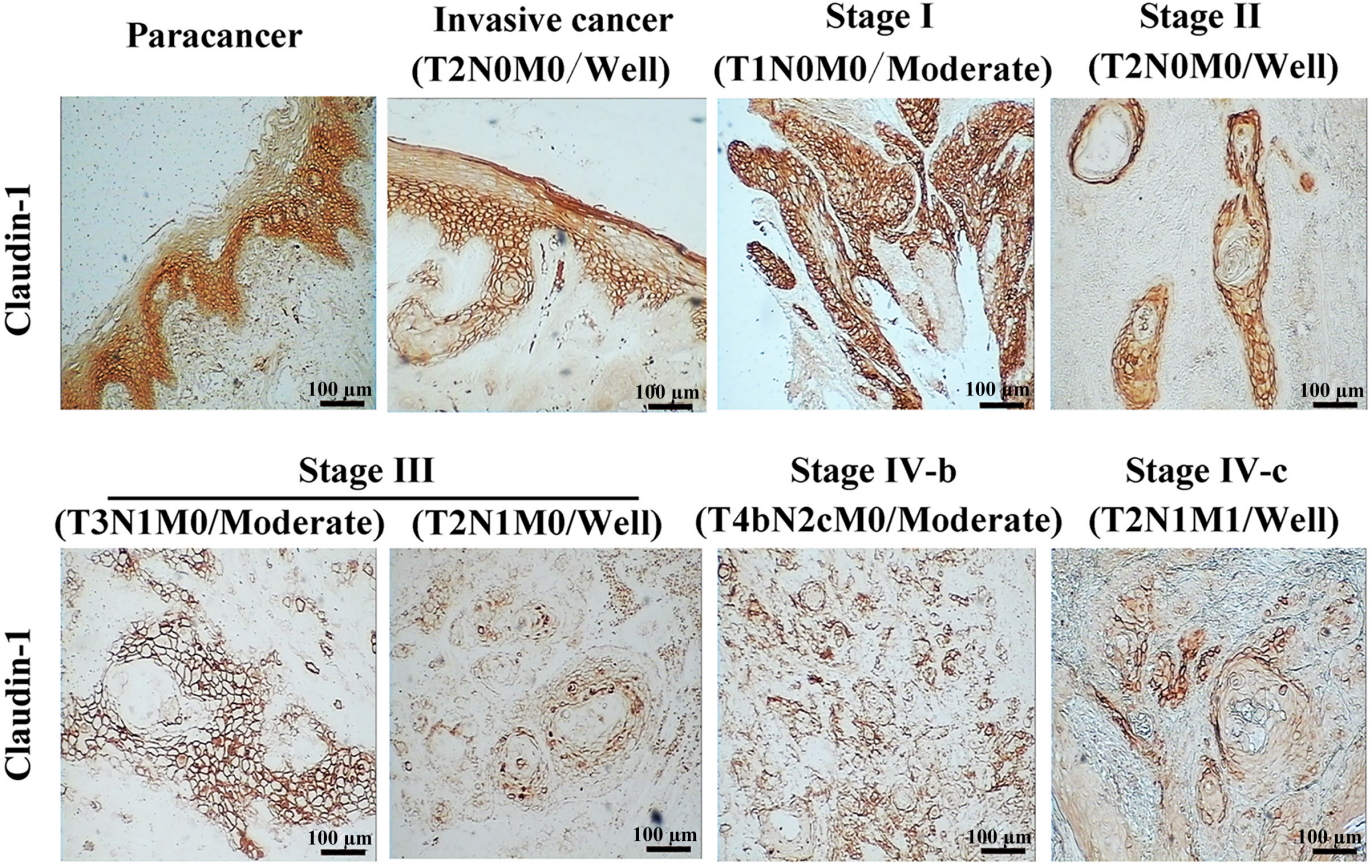
Expression and distribution of Claudin‐1 in tongue tissue. Immunohistochemical staining of Claudin‐1 was performed, and the expression and distribution of Claudin‐1 in the paracancerous tissue, invasive cancer, and cancer in the clinical stages I, II, III, IV‐b, and IV‐c are shown. Bar = 100 μm.

IHC was performed to investigate the expression of Claudin‐1 in TSCC tissues, and the results showed that Claudin‐1 was highly expressed in paracancerous tissues, mainly in the prickle layer, and absent in the basal layer. With the increase in the clinical stage, the expression of Claudin‐1 decreased significantly, especially at the invasive edge of the tongue cancer (Figure 1). Additionally, the relationship between Claudin‐1 expression and the clinical characteristics of TSCC was analyzed (Table 1), and the results showed that Claudin‐1 expression was associated with the clinical T stage (*p* = 0.02) and tumor, node, metastasis (TNM) stages (*p =* 0.04) of TSCC, which indicated that the lower the expression of Claudin‐1, the larger the size of the primary tumor and the later the TNM stage. Nevertheless, there was no association between Claudin‐1 and other clinical features, namely age (*p* = 0.96), sex (*p* = 0.54), tissue type (*p* = 0.93), histological differentiation (*p* = 0.31), lymph node metastasis (*p* = 0.06), and distant metastasis (*p* = 0.87). Clinical analysis indicated the crucial effects of Claudin‐1 loss in tumor growth and deterioration.

**TABLE 1:** Relationship between Claudin‐1 expression and clinical characteristics of TSCC.

Clinical characteristics	Number of patients (%)	Claudin‐1 expression (mean ± SD)	*p*‐Value
Age			0.96
≤60	22 (62)	4.79 ± 2.18	
>60	14 (38)	4.76 ± 2.13	
Sex			0.54
Male	20 (56)	5.03 ± 2.43	
Female	16 (44)	4.59 ± 1.69	
Tissue type			0.93
Paracancer tissues	34	4.64 ± 2.29	
Cancer tissues	36	4.69 ± 2.92	
Histologic differentiation			0.31
Poor	5 (14)	3.92 ± 2.21	
Moderate	11 (31)	4.32 ± 1.99	
Well	20 (55)	5.23 ± 2.03	
Clinical T stage			**0.02**
T1	7 (20)	6.85 ± 2.82	
T2	21 (57)	4.55 ± 1.84	
T3 + T4	8 (23)	3.90 ± 2.09	
Lymph node metastasis			0.06
N0	26 (72)	5.10 ± 2.33	
N1	9 (25)	3.81 ± 0.97	
N2	1 (3)	2.60 ± 0.89	
Distant metastasis			0.87
M0	34 (94)	4.86 ± 2.14	
M1	2 (6)	4.60 ± 3.05	
TNM stage			**0.04**
Stage I	7 (19)	7.26 ± 2.95	
Stage II	15 (42)	4.78 ± 1.86	
Stage III	11 (31)	4.54 ± 2.24	
Stage IV	3 (8)	3.69 ± 0.90	

*Note*: The expression of Clauin‐1 detected by the quantitative analysis of IHC images by the Image Pro Plus 6.0 software and shown as the mean ± SD. *p*‐Values that < 0.05 were showed in bold font to indicate the statistical significance.

Abbreviations: IHC, immunohistochemistry; SD, standard deviation; TNM, tumor, node, metastasis; TSCC, tongue squamous cell carcinoma.

### 3.2 Activation of AMPK increased Claudin‐1 expression and induced its membrane translocation

**FIGURE 2:**
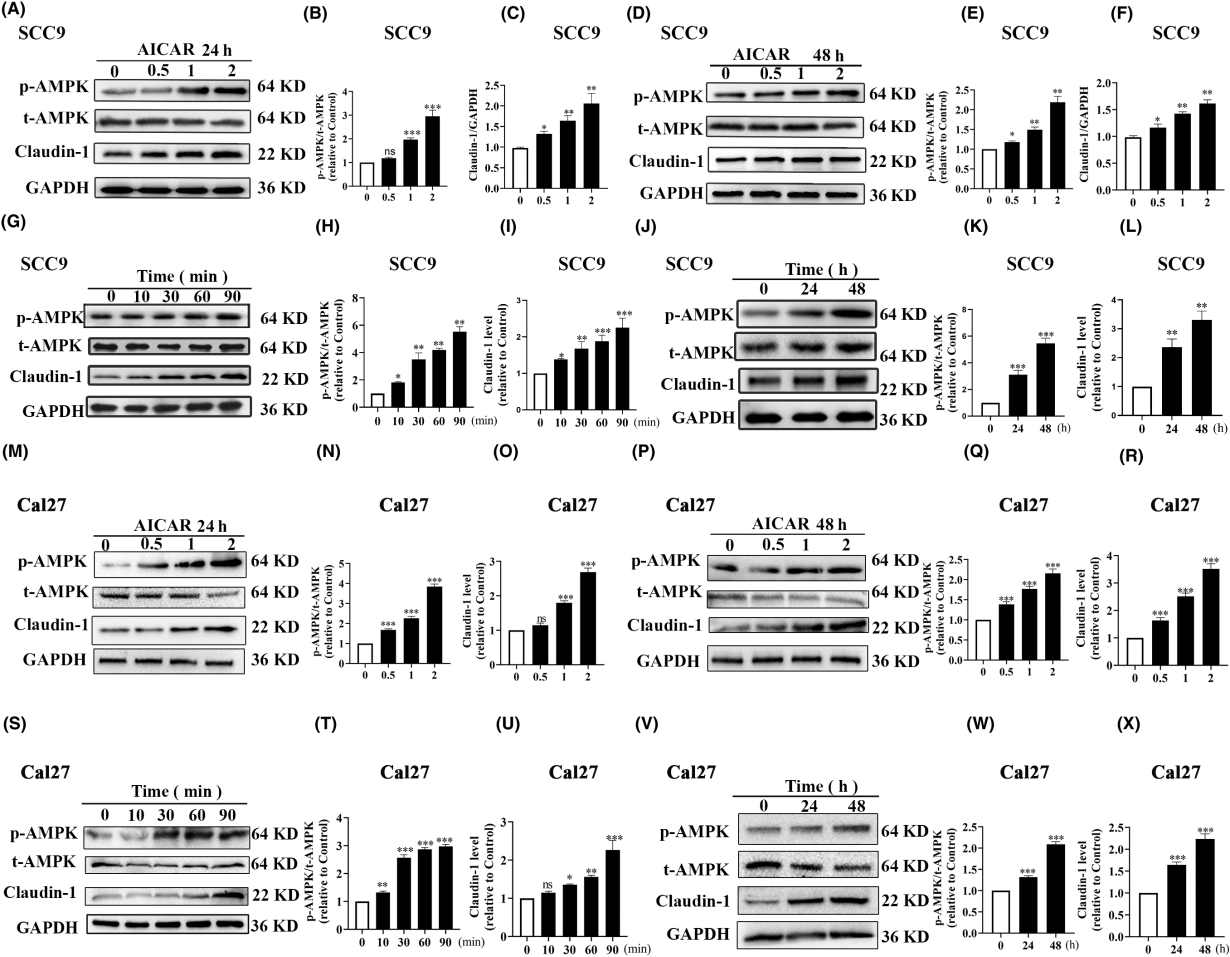
Adenosine monophosphate–activated protein kinase (AMPK) activation can increase Claudin‐1 expression in a time‐dependent manner. The expression of phosphorylated‐AMPK (p‐AMPK), t‐AMPK, and Claudin‐1 in SCC9 cells after treatment with 0.5, 1, or 2 mmol/L 5‐aminoimidazole‐4‐carboxamide1‐β‐d‐ribofuranoside (AICAR) for 24 h (A–C) and 48 h (D–F). SCC9 cells were treated with 1 mmol/L AICAR for a short time for 10, 30, 60, and 90 min (G–I) and a long time for 24 and 48 h (J–L). The expression of p‐AMPK, t‐AMPK, and Claudin‐1 in Cal27 cells after treatment with 0.5, 1, or 2 mmol/L AICAR for 24 h (M–O) and 48 h (P–R). Cal27 cells were treated with 1 mmol/L AICAR for a short time for 10, 30, 60, and 90 min (S–U) and a long time for 24 h and 48 h (V–X). The protein expression was determined through western blotting, with glyceraldehyde 3‐phosphate dehydrogenase (GAPDH) serving as the normalized control. Data are presented as the mean ± standard deviation from three independent experiments. ns, not significant; *p < 0.05; **p < 0.01; and ***p < 0.001.

**FIGURE 3:**
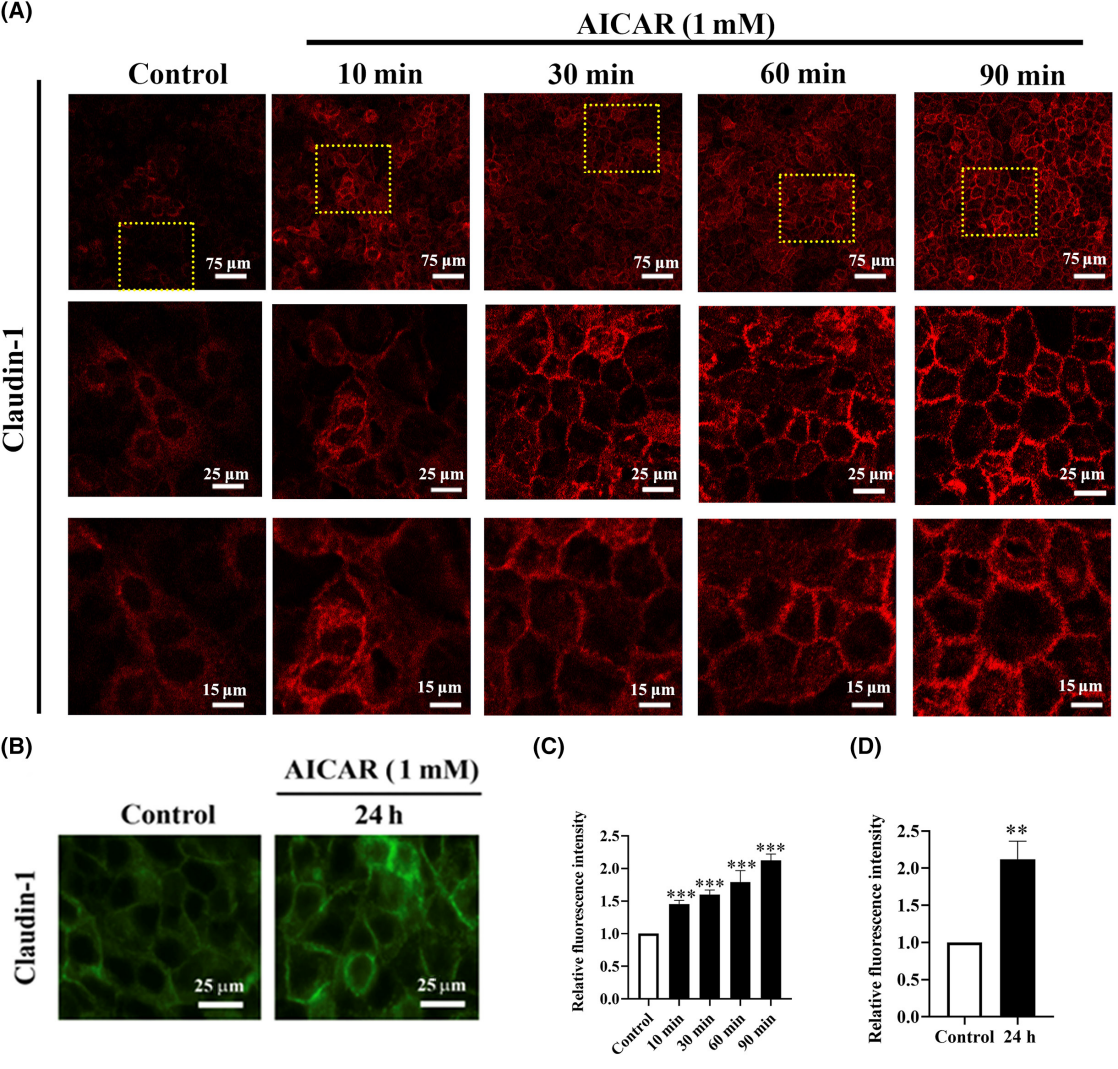
Adenosine monophosphate–activated protein kinase (AMPK) activation can induce Claudin‐1 membranous translocation in a time‐dependent manner. The distribution of Claudin‐1 was examined using immunofluorescence in SCC9 cells treated with 1 mmol/L AICAR for short durations of 10, 30, 60, and 90 min with the same field of view at multiple magnifications (A) and for a longer duration of 24 h (B). Representative images of Claudin‐1 for short‐time treatment (red staining), 24 h treatment (green staining), and their semi‐quantitative results are shown (C, D). Bar = 15, 25, and 75 μm. ns, not significant; **p < 0.01; and ***p < 0.001.

Different concentrations of AICAR (0.5, 1, or 2 mmol/L), the agonist of AMPK, were used to treat SCC9 and Cal27 cells for 24 and 48 h, respectively. The results showed that AICAR stably activated AMPK and increased Claudin‐1 expression in a concentration‐dependent manner in SCC9 (Figure 2A–F) and Cal27 (Figure 2M–R) cells. The effective concentration of 1 mmol/L was used for AICAR treatment in subsequent experiments. After treatment with 1 mmol/L AICAR, Claudin‐1 expression increased within 90 min in both SCC9 (Figure 2G–I) and Cal27 (Figure 2S–U) cells, which remained considerably upregulated after prolonged treatment of 48 h in SCC9 (Figure 2J–L) and Cal27 (Figure 2V–X) cells. Furthermore, the immunofluorescence results showed that the activation of AMPK induced Claudin‐1 translocation from the cytoplasm to the membrane in a time‐dependent manner within 90 min (Figure 3A,C), which persisted for 24 h (Figure 3B,D). These results suggested that AMPK activation increases Claudin‐1 expression and its membrane translocation.

### 3.3 Activation of AMPK inhibits EMT of TSCC cells

**FIGURE 4:**
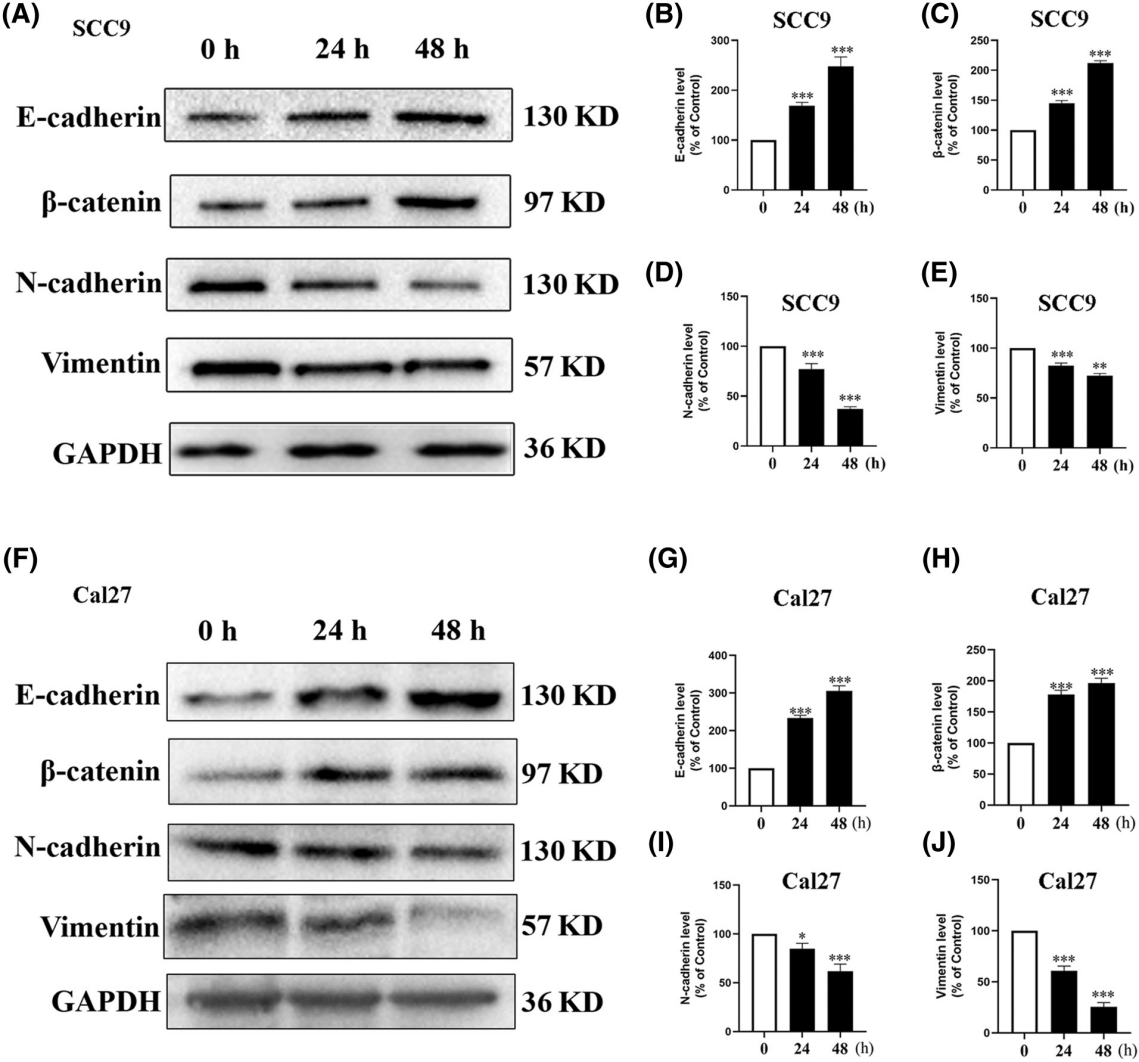
Activation of adenosine monophosphate–activated protein kinase (AMPK) inhibits the epithelial–mesenchymal transition (EMT) of tongue squamous cell carcinoma cells. The expression of EMT‐related proteins, namely E‐cadherin, β‐catenin, N‐cadherin, and vimentin, was detected by western blotting after 5‐aminoimidazole‐4‐carboxamide1‐β‐d‐ribofuranoside (AICAR, 1 mmol/L) treatment for 24 h in SCC9 cells (A–E), and Cal27 cells (F–J). Glyceraldehyde 3‐phosphate dehydrogenase (GAPDH) was used as the normalized control. Data are presented as the mean ± standard deviation from three independent experiments. *p < 0.05; **p < 0.01; and ***p < 0.001.

EMT marks the initiation of tumor migration; therefore, we thoroughly explored the effect of AMPK activation on EMT markers in TSCC cells. The results showed that after AMPK activation by AICAR, the expression of epithelial markers such as E‐cadherin and β‐catenin was upregulated significantly, whereas the expression of mesenchymal markers such as N‐cadherin and vimentin was downregulated to varying degrees in both SCC9 (Figure 4A–E) and Cal27 (Figure 4F–J) cells. These results suggest that the activation of AMPK might inhibit the migration of TSCC cells via the inhibition of EMT.

### 3.4 Knockdown of Claudin‐1 promoted EMT and migration of SCC9 cells

**FIGURE 5:**
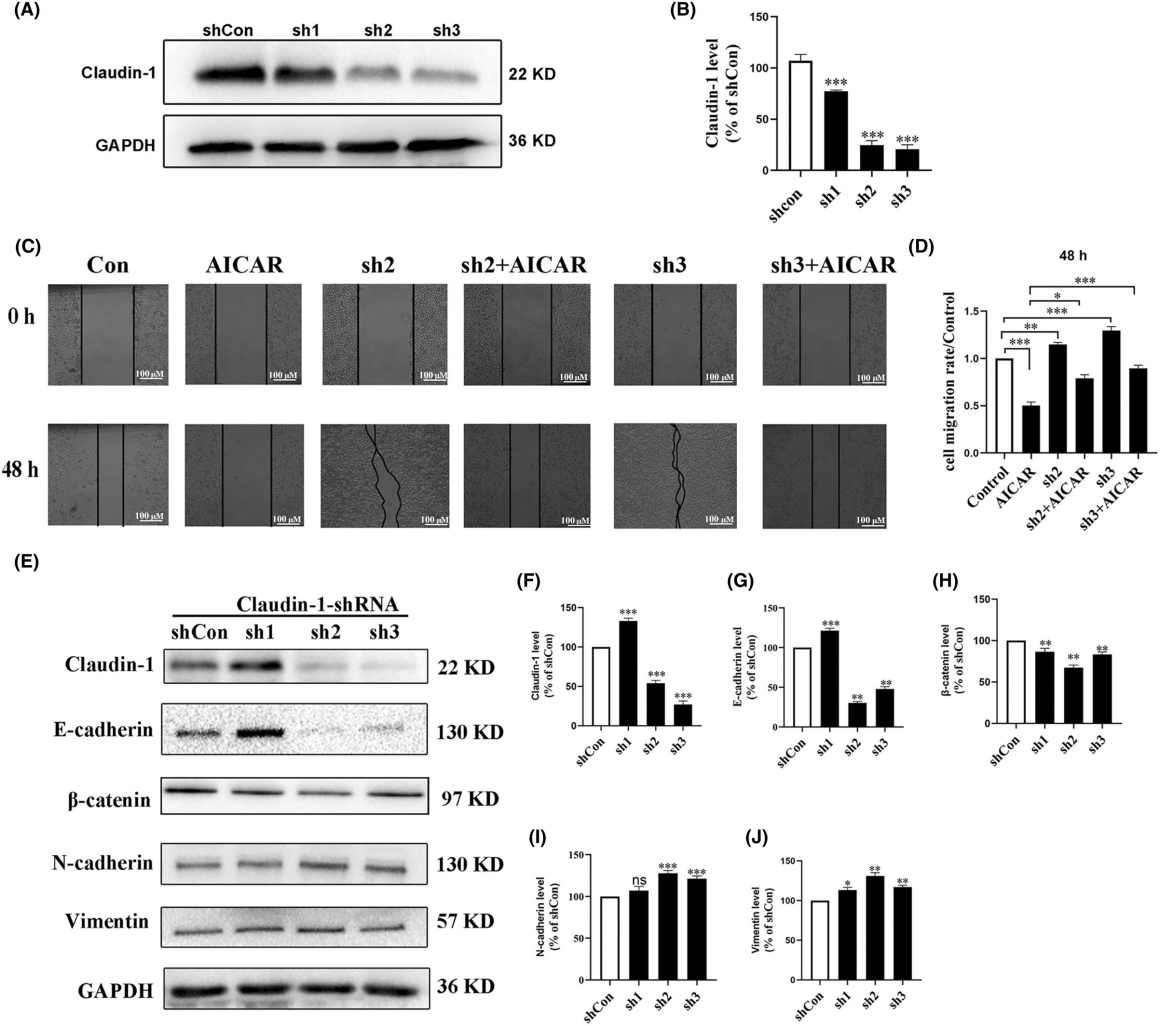
Knockdown of Claudin‐1 promoted the epithelial–mesenchymal transition (EMT) and migration of SCC9 cells. (A, B) The protein expression of Claudin‐1 in SCC9 cells transfected with short hairpin RNAs (shRNAs; namely sh1, sh2, and sh3) for 72 h was detected by western blotting. (C, D) Cell migration was assessed through the wound healing assay after incubation with or without 5‐aminoimidazole‐4‐carboxamide1‐β‐D‐ribofuranoside (AICAR, 1 mmol/L) for 48 h in Claudin‐1 stable knockdown cells transfected with Claudin‐1 shRNA (sh2 or sh3) and parent SCC9 cells. The relative migration effect of the cells was measured by the mean wound healing area. Data are presented as the mean ± standard deviation of four separate fields from three independent experiments. *p < 0.05; **p < 0.01; and ***p < 0.001. (E–J) The EMT‐related protein expression in Claudin‐1 stable knockdown cell lines was detected by western blotting. All the results of western blotting are represented by three independent experiments. Glyceraldehyde 3‐phosphate dehydrogenase (GAPDH) was used as the normalized control. shCon, negative control; ns, not significant; *p < 0.05; **p < 0.01; and ***p < 0.001.

To determine if Claudin‐1 was associated with EMT and the migration of TSCC cells, three Claudin‐1 shRNAs (namely sh1, sh2, and sh3) were used and Claudin‐1 stable knockdown cell lines were constructed using SCC9 cells. As the protein expression of Claudin‐1 was markedly downregulated by sh2 and sh3 interference (Figure 5A,B), they were used for the subsequent experiments. A wound healing assay was performed, which revealed that cell migration was inhibited after AMPK activation and was promoted after the knockdown of Claudin‐1 within 48 h, indicating that the inhibitory effect of AMPK on cell migration was attenuated when Claudin‐1 was stably knocked down (Figure 5C,D). In addition, we found that the expression of E‐cadherin and β‐catenin was markedly downregulated after the knockdown of Claudin‐1, whereas the expression of N‐cadherin and vimentin was notably upregulated (Figure 5E–J), which revealed that the loss of Claudin‐1 could accelerate the migration of SCC9 cells by promoting EMT.

### 3.5 The inhibitory effect of AMPK on EMT and migration was mediated by Claudin‐1

**FIGURE 6:**
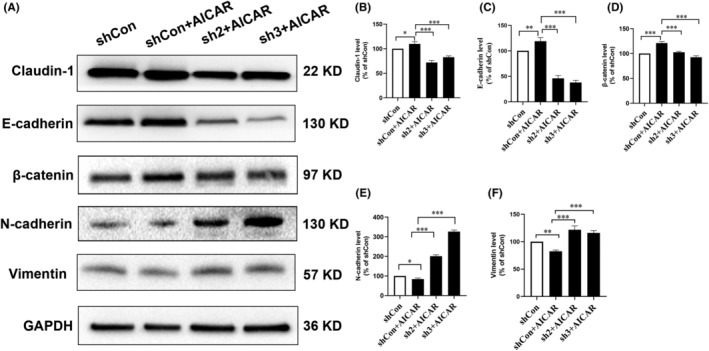
The inhibitory effect of adenosine monophosphate–activated protein kinase on epithelial–mesenchymal transition (EMT) and migration were mediated by Claudin‐1. (A–F) The EMT‐related protein expression was detected by western blotting after 5‐aminoimidazole‐4‐carboxamide1‐β‐d‐ribofuranoside (AICAR, 1 mmol/L) treatment for 24 h in Claudin‐1 stable knockdown cells transfected with Claudin‐1 short hairpin RNA (sh2 or sh3). The results are represented by three independent experiments. Glyceraldehyde 3‐phosphate dehydrogenase (GAPDH) was used as the normalized control. shCon: negative control; *p < 0.05; **p < 0.01; and ***p < 0.001.

To investigate whether Claudin‐1 is involved in the migratory inhibitory impact of AMPK via the suppression of EMT, we assessed the levels of EMT‐associated proteins in Claudin‐1 stable knockdown cell lines following AICAR treatment. The levels of E‐cadherin and β‐catenin displayed a decrease, whereas N‐cadherin and vimentin levels showed an increase (Figure 6A–F). These significant alterations in the levels of EMT markers suggest that Claudin‐1 plays a crucial role in the EMT process of SCC9 cells and may reverse the AMPK activation–induced inhibition of EMT.

## 4 DISCUSSION

Over the past decade, studies have found that abnormalities in TJ function and expression are associated with tumor progression. Claudin‐1 is a major membrane protein found in TJs.^23^ Several studies have investigated the expression and function of Claudin‐1 in SCC or TSCC cells; However, its precise role as a tumor promoter, suppressor, or both, in SCC and TSCC cells remains unclear. For example, in HNSC and OSCC, Claudin‐1 is highly expressed in tumor tissues compared with that in normal tissues.^15^, ^24^ In HNSC, Claudin‐1 acted as a tumor promoter, and the knockdown of Claudin‐1 inhibited the invasive phenotype by downregulating EMT.^24^ In the present study, the immunohistochemical staining of Claudin‐1 showed its high expression in paracancerous tissues, whereas in tumor tissues, the expression of Claudin‐1 decreased considerably with an increase in the clinical stage, especially in the invasive edge of the tongue cancer. Our results are consistent with previous studies that found that Claudin‐1 expression was significantly positive on the cell membrane but negative in the basal layer of normal oral tissues,^25^ whereas in tumor, Claudin‐1 expression decreased in the invasion front and negative in TSCC tissues without vascular infiltration.^26^ Moreover, we found that the lower the Claudin‐1 expression, the larger the tumor size and the later the TNM stage, which suggested a potential relationship between Claudin‐1 loss and tumor deterioration. Additionally, in our study, we discovered that Claudin‐1 functions as a tumor suppressor in TSCC cells; knockdown of Claudin‐1 promoted EMT and accelerated the migration of SCC9 cells, which is consistent with previous studies that the loss of Claudin‐1 from the membrane via endocytosis promoted cell migration in TSCC.^14^ Our findings are consistent with the results of previous studies showing that Claudin‐1 is involved in tumor progression in TSCC.^25^

In the present study, we used the UALCAN database (http://ualcan.path.uab.edu/) to analyze the expression of Claudin‐1 in HNSC. Surprisingly, Claudin‐1 mRNA in HNSC was highly expressed in primary tumor tissues and various individual cancer stages than in normal tissues (Figure S1a,b). These results are inconsistent with our IHC results of Claudin‐1 protein expression, which showed a significant decrease in Claudin‐1 expression with the increase in the clinical stages of TSCC (*p* = 0.04), although no significant difference was found between the paracancerous and cancerous group. We attempted to explain this paradox and speculated that the differences in the sample might be an important reason. The samples from the UALCAN database were Claudin‐1 mRNA from HNSC, which includes multiple cancers in the paranasal sinuses, nasal cavity, oral cavity, tongue, salivary glands, larynx, and pharynx, whereas the samples in our results were Claudin‐1 protein from TSCC only. The paracancerous tissues in our study were collected from sites at least 2 cm away from the tumor mass, which was distinct from the normal group in the UALCAN database. However, due to the specificities of the tumor microenvironment, mRNA and protein expression may differ significantly depending on the rate of protein degradation, post‐translational modification, and negative feedback regulation. Furthermore, based on the race of the patient, we found that patients from Asia (*n* = 11) only accounted for a small percentage of total patients in the UALCAN database, whereas patients from Caucasian (*n* = 444) and African American (*n* = 47) origins were the majority (Figure S1c). Notably, the Caucasian and African American groups showed a significant increase in Claudin‐1 mRNA levels; However, no significant difference was found between the Asian and normal groups. Additionally, a downward trend in Claudin‐1 mRNA levels was observed in the tumor tissues based on nodal metastasis status. The median decreased from 115 in the N0 stage to 80 in the N3 stage. Significantly, there was a decrease from 115 in the N0 stage to 96 in the N1 stage (Figure S1d). These results are consistent with our study and suggest that lower expression of Claudin‐1 is associated with more aggressive tumors.

AMPK activation promotes TJ assembly and polarity re‐establishment; however, changes in TJ function can alter tumor progression.^19^, ^27^ Our previous study showed that AMPK activation could inhibit cell migration by disrupting the TJ structure mediated by ZO‐1 in TSCC.^20^ Previous studies have reported that the overexpression of Claudin‐1 could inhibit the migration and invasion of TSCC cells.^28^ Based on previous studies, we first explored whether the activation of AMPK by AICAR could increase the expression and induce the membranous translocation of Claudin‐1, thus inhibiting the migration and EMT of TSCC cells.

EMT is associated with tumorigenesis, invasion, metastasis, and resistance to chemoradiotherapy.^16^ The loss of cell–cell adhesions, such as TJs, promotes EMT and induces cancer cell invasion and metastasis.^29^ Our results showed that AMPK activation significantly inhibited EMT in TSCC cells. Furthermore, we revealed that the knockdown of Claudin‐1 promotes EMT and reverses the inhibition of EMT induced by AMPK activation, suggesting that the inhibitory effect of AMPK on EMT and migration is mediated by Claudin‐1.

## 5 CONCLUSIONS

In conclusion, we show that Claudin‐1 levels in TSCC are correlated with tumor TNM stages and primary foci size, suggesting that Claudin‐1 expression is related to tumor malignancy.The activation of AMPK could inhibit the migration of TSCC by targeting Claudin‐1 via suppression of EMT.

## AUTHOR CONTRIBUTIONS

Xin‐Yue Zhou wrote the manuscript, Xin‐Yue Zhou and Qiu‐Ming Liu performed the experiments, Zhuang Li, Xia‐Yang Liu, Qi‐Wei Zhao, and Yu Wang participated in the experiments and assisted with the analysis. Feng‐Hua Wu and Gang Zhao designed the research and helped interpret the data. Xiao‐Hong Guo and Rui Sun acquired the fundings and supervised the research. All the authors have read and approved the final manuscript.

## FUNDING INFORMATION

This work was supported by grants from National Natural Science Foundation of China (no.: 82174020 and no.: 31301137), Shanxi Basic Research Program of China (202103021224378), and Shanxi Bethune Hospital Talent Introduction Research Start‐up Fund of China (2022RC13).

## CONFLICT OF INTEREST STATEMENT

The authors have no competing interests to declare that are relevant to the content of this article.

## ETHICS STATEMENT

This clinical study was approved by the Ethics Committee of the Peking University Health Science Center (IRB00001052‐09087).

## PATIENT CONSENT STATEMENT

All patients provided written informed consent before participating in the study.

## Supporting information


Figure S1

## Data Availability

The datasets used or analyzed during the current study are available from the corresponding author on reasonable request. The authors did not use the AI tools to analyze and draw insights from data as part of the research process.
