# Supplementary figures and images for: The activation of adenosine monophosphate–activated protein kinase inhibits the migration of tongue squamous cell carcinoma cells by targeting Claudin‐1 via epithelial–mesenchymal transition
Source: Animal Model Exp Med. 2024 Jul 17;7(5):606–16. doi: 10.1002/ame2.12444 (PMC11528389; doi:10.1002/ame2.12444)

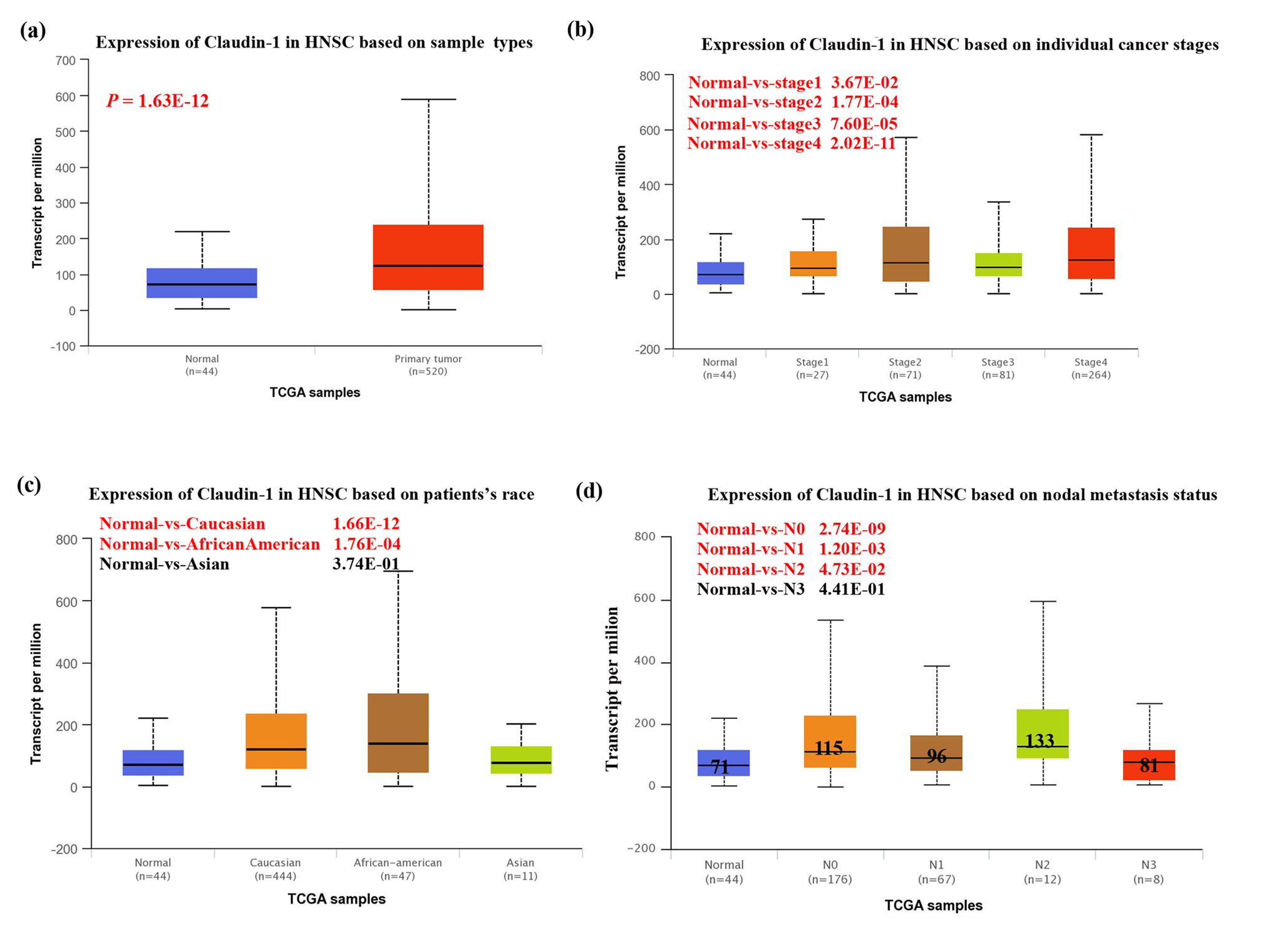

Supplement: Supplementary file 1 — Figure S1 [file AME2-7-606-s001.tif]
